# Predicting Uncertain Multi-Dimensional Adulthood Outcomes From Childhood and Adolescent Data in People Referred to Autism Services

**DOI:** 10.3389/fpsyg.2021.594462

**Published:** 2021-02-09

**Authors:** Gordon Forbes, Catherine Lord, Rebecca Elias, Andrew Pickles

**Affiliations:** ^1^Department of Biostatistics and Health Informatics, Institute of Psychiatry, Psychology and Neuroscience, King’s College London, London, United Kingdom; ^2^Department of Psychiatry, University of California, Los Angeles, Los Angeles, CA, United States

**Keywords:** autism spectrum disorder, adult outcomes, early diagnosis cohort, childhood, prediction

## Abstract

**Introduction:**

Autism spectrum disorder is a highly heterogeneous diagnosis. When a child is referred to autism services or receives a diagnosis of autism spectrum disorder it is not known what their potential adult outcomes could be. We consider the challenge of making predictions of an individual child’s long-term multi-facetted adult outcome, focussing on which aspects are predictable and which are not.

**Methods:**

We used data from 123 adults participating in the Autism Early Diagnosis Cohort. Participants were recruited from age 2 and followed up repeatedly through childhood and adolescence to adulthood. We predicted 14 adult outcome measures including cognitive, behavioral and well-being measures. Continuous outcomes were modeled using lasso regression and ordinal outcomes were modeled using proportional odds regression. Optimism corrected predictive performance was calculated using cross-validation or bootstrap. We also illustrated the prediction of an overall composite formed by weighting outcome measures by priorities elicited from parents.

**Results:**

We found good predictive performance from age 9 for verbal and non-verbal IQ, and daily living skills. Predictions for symptom severity, hyperactivity and irritability improved with inclusion of behavioral data collected in adolescence but remained modest. For other outcomes covering well-being, depression, and positive and negative affect we found no ability to predict adult outcomes at any age. Predictions of composites based on parental priorities differed in magnitude and precision depending on which parts of the adult outcome were given more weight.

**Conclusion:**

Verbal and non-verbal IQ, and daily living skills can be predicted well from assessments made in childhood. For other adult outcomes, it is challenging to make meaningful predictions from assessments made in childhood and adolescence using the measures employed in this study. Future work should replicate and validate the present findings in different samples, investigate whether the availability of different measures in childhood and adolescence can improve predictions, and consider systematic differences in priorities.

## Introduction

Autism spectrum disorder (ASD) is a neurodevelopmental disorder, commonly diagnosed in early childhood and generally thought to have a lifelong impact. It is, however, highly heterogeneous, both among those assessed at any one time and in how individuals develop over time (Howlin et al., 2000; Seltzer et al., 2004; Billstedt et al., 2011; Bishop-Fitzpatrick et al., 2016; Simonoff et al., 2019; Stringer et al., 2020). Given the considerable heterogeneity, it would be clinically helpful for both parents of autistic children and the clinicians they work with to have a clear understanding of what potential adult outcomes could be; both what might be expected with some confidence and where it would be premature to begin to form any expectation. This can be particularly important for individual planning, both in terms of where intervention or support may be required and also to anticipate the potential financial impact of autism, which may be considerable (Buescher et al., 2014). At present, determining prognosis for a young autistic child is a difficult task. Clinicians often rely on translating clinical research which provides meaningful and relevant predictive factors, understanding of average adult outcomes for groups with different characteristics, and important insights gained through the clinician’s experience. Additionally, individual opportunities, experiences, and preferences are considered. Limited information is available to guide clinicians and families to determine how the development and phenotypic expression of their child may deviate from other autistic children.

The developmental nature of ASD means that the process of sketching out a long-term prognosis is not a one-time exercise, but one that is refined as a child matures and as the span and depth of measurement increases. This task is made more complicated by the multi-facetted nature of the adult outcome and the increased variety of contexts and resources available to autistic adults, compared to the more structured settings available in childhood. What constitutes a good outcome in adulthood for an autistic child can vary from person to person (Lounds Taylor, 2017; McCauley et al., 2020). Reduction of the severity of symptoms related to ASD can be viewed as both positive or negative (Bagatell, 2010). Poor social functioning has been observed in autistic adults and may lead to reduced quality of life (McCauley et al., 2020) but not always (Billstedt et al., 2011; Howlin et al., 2013). To make prognoses that can capture what a good outcome may look like, we take two approaches. Firstly, we a consider a diverse set of measures to describe outcomes in adulthood. Secondly, we look to create a personalized composite outcome based on the priorities of an individual parent.

This study is one of a series exploring the methods and scope for undertaking individual level outcome prediction for developmental processes. This analysis is based on the Autism Early Diagnosis Cohort (EDX) (Lord et al., 2006, 2020) of children referred for possible autism when aged 2–3 years old. Our previous work using this data has involved grouping participants either using latent class modeling or groups defined by a-priori cut offs IQ and autism diagnosis status (Lord et al., 2020). Latent class modeling is a statistical technique which forms groups of people with similar outcomes, or trajectories of outcomes, across a range of measures. We used a latent class model to reduce the adult outcome, characterized by 15 diverse measures spanning IQ to well-being, to a set of four classes each with a distinctive profile across these measures (Pickles et al., 2020). A second latent class approach to this data involved creating groups based on trajectories of ADHD, anxiety and depression symptoms (McCauley et al., 2020). These different approaches have led to important insights. For example, prediction of latent classes formed from the adult outcome was possible using, in addition to socio-demographic variables, measures of ASD, IQ and a composite of simple functional skills, taken at approximately ages 2, 3, 5, and 9 years of age. The latent classes, however, were heavily influenced by a relatively small subset of easily predicted measures that had been included in the full set of adult-outcome measures. Moreover, parents, clinicians and patients may place greater or lesser importance on different facets of the outcome, and this pattern of relative weight attached to each measure may differ within each of these groups.

This study extends previous work by using a quite different strategy and methods, exploring the impact of extending the span of measures available for prediction into more behavioral and emotional problem domains, and considering prediction from a little closer to the adult outcome by including measures taken beyond childhood and into adolescence. We focus on the prediction of the individual adult outcome measures using regression modeling, which has not been considered in this data before, and then the prediction of composites formed from weighting predictions of individual measures. A notable issue in prediction modeling is the potential for apparent model performance to grossly exaggerate predictive performance in a new sample (Steyerberg and Harrell, 2016). This can be a particular issue when sample sizes are small. To compensate for this we use resampling techniques (bootstrap and cross validation), commonly used in prediction modeling, to provide estimates for model performance taking into account the optimism in apparent measures of model performance (Steyerberg, 2019). A second, related challenge, is that overfitting of the data can lead to poor model performance in new samples. To reduce the risk of overfitting we use LASSO regression, which shrinks parameter estimates toward zero (no association), to prevent associations in the data that exist by chance being modeled (Friedman et al., 2010). Another challenge when predicting outcomes that are measured with some error is that the reliability of the measure can act as a ceiling to predictive performance. Alongside our modeling results we present the limits of model performance that can be expected from reported test-retest reliability of the measure we employ. This allows an assessment to be made as to where we are close to the possible limits of prediction and where it could be possible for improved predictions to be made.

We demonstrate the use of composite outcomes using priorities obtained from parents of autistic children, not involved in the Autism Early Diagnosis Cohort. For this preliminary work, priorities were obtained from parents, rather than the young autistic people directly. The approach could equally accommodate priorities obtained from the individual themselves. This study differs from earlier work examining predictors of adult outcomes (Magiati et al., 2014; Zimmerman et al., 2018) as we examine the extent to which different measures in the adult outcome can be predicted, rather than which variables are predictors. This work is intended to help frame discussions between clinicians, carers and autistic individuals as to plans and priorities, hopes and evidence-based expectations.

The prediction of individual adult outcome measures may provide insight into measures that could better predict facets of their outcome in adulthood that are currently predicted poorly. It allows us to better identify those aspects of the outcome that are likely determined by early childhood or those predictable only by measures taken in late adolescence. We also identify outcomes that are simply unpredictable, perhaps due to their episodic nature, unreliable measurement, or outcomes which are highly variable due to a large impact of unmeasured, or unobservable, biological or environmental factors.

The potential for developing a prediction tool also raises questions as to the context in which such a tool might be used. This may include the developmental stage of the child, the readiness of the child and parents for information, or the choices they have available or need to make. Incorporating in predictions the autistic individuals’ priorities, or their parents priorities, may help reflect this context. Discussion of the clinical implementation of prediction tools requires knowledge of what is and what is not predictable and by when. This study provides a starting point. Work in this area may also be relevant for prioritizing interventions for an individual, or for the development of new interventions, as well as the defining and selection of outcome measures in intervention studies.

## Materials and Methods

### Participants

This analysis uses data from the Autism Early Diagnosis Cohort (Lord et al., 2006). The Autism Early Diagnosis Cohort enrolled 192 participants from North Carolina and Chicago referred for possible autism between age 2 and 3, and 21 referred to the autism program as exhibiting developmental delay. A further 31 participants were recruited from similar sources in Michigan at age 9, with the intention of increasing the sample size for subsequent follow ups. Families and, later, participants (where possible) provided informed consent. The research was approved by institutional review boards from Weill Cornell Medicine, the University of Michigan and UCLA.

**FIGURE 1:**
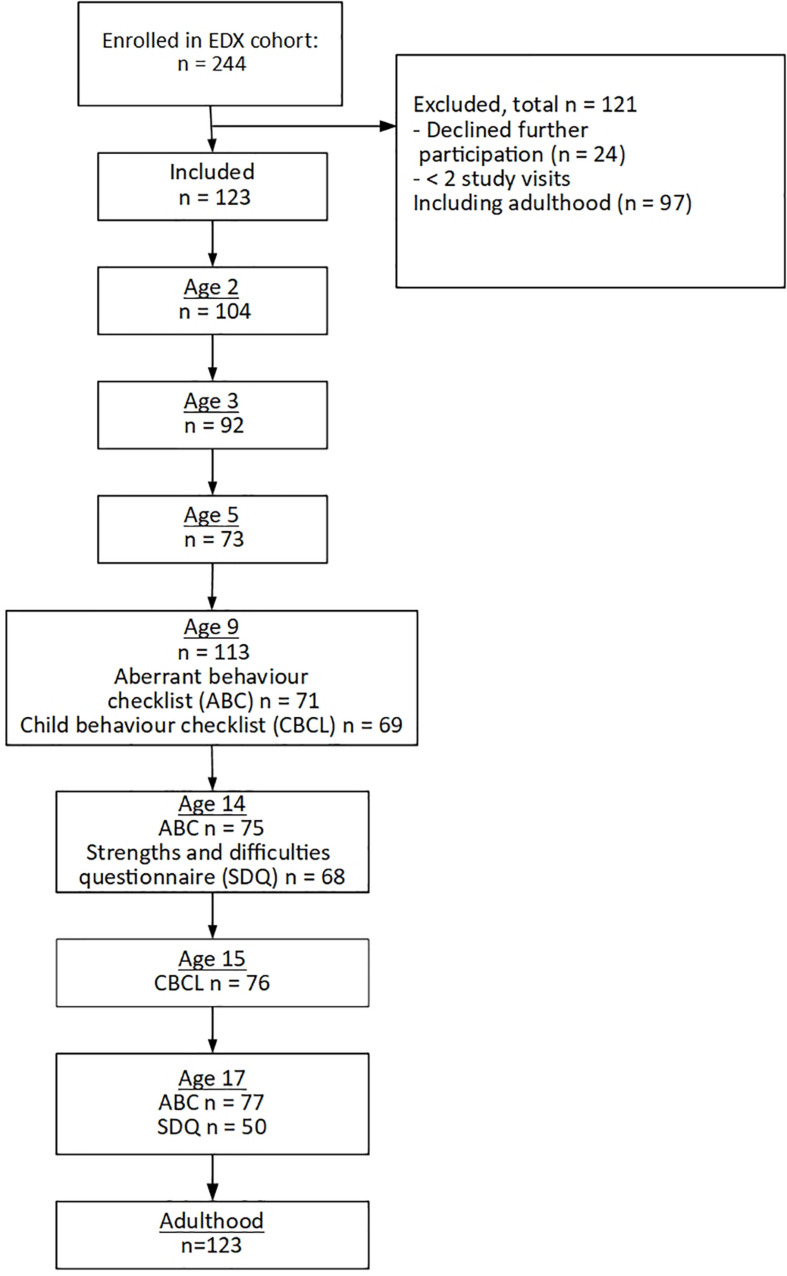
Participant flow through the study.

The analysis presented in this paper includes the 123 young adults who participated in at least one childhood assessment, and one assessment in adulthood (Figure 1). Loss to follow up occurred predominantly due to geographical relocation or losing contact. Twenty four participants (11.3%) declined ongoing participation and were excluded from the analysis. Loss to follow up was associated with race and parental education, with drop out higher for African-Americans and families with the lowest educational levels (Pickles et al., 2020).

### Measures and Data Collection

Face to face assessments were undertaken with children and their parents at ages 2, 3, 5 (North Carolina only), 9, 19, 21 (a subset of participants), and 25 years. Further telephone interviews were conducted at age 14, 15, and 17. Assessments were carried out by a team of researchers who had achieved research reliability on the measures administered, led by a Ph.D. level psychologist.

At ages 2–9, and adulthood the severity of autism symptoms was measured using the Calibrated Severity Scores (CSS), calculated from the Autism Diagnostic Observation Schedule (ADOS) (Gotham et al., 2009); Verbal and non-verbal IQ were measured using the Wechsler Abbreviated Scale of Intelligence (Wechsler, 1999), Differential Ability Scales (Elliott, 2007) and the Mullen Scales of Early Learning (Mullen, 1995). Daily living skills were measured using the Daily Living Standard score from the Vineland Adaptive Behavior Scales (Sparrow et al., 2005). From age 9, irritability and hyperactivity were measured using subscales of the Aberrant Behavior Checklist (Aman and Singh, 1994); For an overall measure of behavioral problems we used the total problem score taken from the Child Behavior Checklist (CBCL) (Achenbach and Rescorla, 2001) and Adult behavior Checklist (ABCL) (Achenbach and Rescorla, 2003). The Strengths and Difficulties Questionnaire (SDQ) (Goodman, 1997) was completed by teachers about the children in the study at age 14 and 17. In addition to measures used in childhood, adult assessments included the positive and negative subscales from the Positive and Negative Affect Schedule (PANAS-P and PANAS-N) (Watson et al., 1988), the Beck Depression Inventory-II (BDI-II) (Beck et al., 1996); the Well-being Questionnaire (WBQ) (Ryff, 1989); and ordinal assessments of participant’s living and work and friendships were made using the Social and Emotional Functioning Interview (SEF-I and SEF-S) (Rutter et al., 1988). Partially completed scales were pro-rated when 80% of items were completed. More details on the measures used and the schedule of assessment are given in Supplementary Tables 1, 2.

### Eliciting Parent Priorities

To demonstrate how different priorities across outcomes could lead to differences in predictions, we provide predictions weighted with the priorities of two parents of autistic children consulted as part of a parent involvement meeting, arranged as part of a separate study, the priorities for these two parents are labeled parent A and parent B. Questionnaires were completed independently prior to the meeting taking place. Priorities were obtained using a questionnaire which asked parents to allocate points, up to a total of 100, across 10 facets of a child’s adult outcome which were then mapped to the outcomes collected in the study (Supplementary Tables 3, 4). These priorities are intended to illustrate our approach, not for inferences about more widespread parental priorities.

### Sample Size

The available sample size varies across outcomes from 123 for verbal and non-verbal IQ to 91 for the well-being questionnaire. The required sample size for the development of prediction models depends on the number of predictors included in the model and the value of r-squared (Riley et al., 2019). Using Riley et al.’s criteria for linear regression models with 10 predictors (the numbers of predictor we use for models at ages 2–5), and 123 observations the recommended minimum r-squared to reduce the risk of over fitting is 0.44, with 91 outcomes observed outcomes the minimum r-squared is 0.55. For linear regression models fit with 13 predictors (as fitted using post age 9 predictors) minimum r-squared ranges from 0.55 to 0.66 for the available sample size. When the emotion and prosocial subscales of the SDQ are additionally included the minimum r-squared is from 0.6 to 0.7. These r-squared values can be used to guide interpretation of our results, indicating where models may be at risk of overfitting, which may lead to over-optimistic estimates of predictive performance (r-squared).

### Statistical Analysis

For the assessment of predictive performance, statistical analysis was conducted using linear regression for continuous outcomes and proportional odds logistic regression for ordinal outcomes. A separate model was fit for each outcome and at each time point. The analysis for continuous outcomes was repeated using LASSO regression implemented in R using the glmnet package (Friedman et al., 2010), with the tuning parameter selected to minimize the mean squared error using leave-one-out cross-validation. The analysis of ordinal outcomes was not repeated using penalized regression as the low number of events in some categories made it unfeasible to estimate the tuning parameter using cross-validation. Apparent assessments of predictive performance are known to be optimistic as the same data is used to assess predictions as is used to fit the models. To compensate for this, we used leave-one-out cross-validation for continuous outcomes and bootstrap (40 repetitions) for ordinal outcomes (Harrell, 2015). For continuous outcomes predictive performance was measured using R-squared. For ordinal outcomes performance was measured using the generalized c-statistic (Harrell, 2015).

At each time point, the same set of predictors were used for all outcomes. At all ages, models included gender, race, and mothers’ education. At ages 2–9, models also included measures from the child’s respective age, including verbal and non-verbal IQ, autism symptom severity, daily living skills, and whether the child had a current diagnosis of autism; models beyond age 9 included these variables measures at age 9 as no further assessments of these measures were made before adulthood. Starting at age 9 irritability, hyperactivity, and behavior problems measured using the CBCL were included in the models with new assessments becoming available at ages 14 (age 15 for CBCL) and 17. At each timepoint only the most up to date measurements of predictors were included in the model. Additional models were fit at age 14 and 17 including the pro-social and emotion subscales from the SDQ. Supplementary Table 5 lists the predictors included at each timepoint.

### Prediction Intervals for Weighted Sums of Outcomes

A composite outcome (a single outcome combining all of the adulthood measures) was formed incorporating individual parent priorities. The composite outcomes were calculated by weighting each component by the priority placed on it, then adding together all the weighted components. Prior to summing, outcomes were standardized to have mean zero, and variance one, and where necessary reverse scored so that positive outcomes indicate a more severe impact of autism. Construction of prediction intervals requires consideration of residual standard deviations in addition to standard errors of parameters (Gelman and Hill, 2006). To allow estimation of prediction intervals for weighted combinations of outcomes correlations between residuals for different outcomes needed to be estimated. To estimate these correlations, we used a structural equation model to jointly model all outcomes, with residuals for all outcomes set to be correlated. Standard errors of prediction were calculated for weighted combinations of outcomes, and prediction intervals were calculated assuming outcomes were normally distributed, or for ordinal outcomes, a normal underlying variable of a probit model. Estimation was conducted using weighted least squares, implemented in R using the Lavaan package (Rosseel, 2012).

Missing data in predictors were imputed using k nearest-neighbor imputation, using 5 neighbors (Dahl, 2007). Imputation of missing data was conducted on the whole dataset, prior to splitting for internal validation. In analysis models for single outcomes (those used to assess predictive performance), participants with missing data for an outcome were excluded from the analysis of that outcome (von Hippel, 2007). For joint models, missing outcomes were imputed in the same way as missing data on predictors.

## Results

Figure 1 shows the numbers followed up at each time point, and numbers for particular measures when not all participants completed the measure. Table 1 gives descriptive statistics for predictors measured at enrolment, behavioral measures at age 14, and adulthood outcomes. Summary statistics for all variables, at all timepoints can be found in Supplementary Table 6.

**TABLE 1:** Summary statistics for first measure of predictors and outcomes.

Measure	*N*	Median (IQR)/*N* (%)	Range
**At recruitment**			
Age	123	2.6 (2.2, 2.9)	1.3–11.8
Female—*N* (%)	123	21 (17%)	0–1
Non-Caucasian—*N* (%)	123	21 (17%)	0–1
Maternal education	123	2 (1, 3)	1–5
Autism symptom severity (CSS)	121	7 (3, 9)	1–10
Verbal IQ	123	37 (23, 60)	10–128
Non-verbal IQ	123	75 (54, 85)	13–132
Daily living skills	106	68.5 (61.2, 74)	52–99
**Outcomes age 14**			
Hyperactivity	75	8 (3.5, 15)	0–31
Irritability	75	5 (1, 13)	0–29
**Adult outcomes**			
Autism symptom severity (CSS)	118	6 (3, 7)	1–10
Verbal IQ	123	46 (20, 103.5)	2–139
Non-verbal IQ	123	72 (26, 105)	3–133
Daily living skills	123	61 (35.5, 78)	17–112
Hyperactivity	104	4.7 (1.5, 11.6)	0–30.8
Irritability	104	4.7 (1, 9.7)	0–37.5
Behavioral problems (ABCL)	94	53 (48, 57)	25–77
Well-being questionnaire	91	189 (169, 208)	134–248
PANAS-P	92	28 (22.8, 33.5)	12–45.5
PANAS-N	93	15 (12, 21)	10–35.5
Depression (BDI)	92	2.5 (0.5, 7.1)	0–30
Independent living	123	2 (2, 2)	1–3
SEF-I friends	106	2 (0.2, 3)	0–3
SEF-I work	113	4 (2, 6)	1–7

**FIGURE 2:**
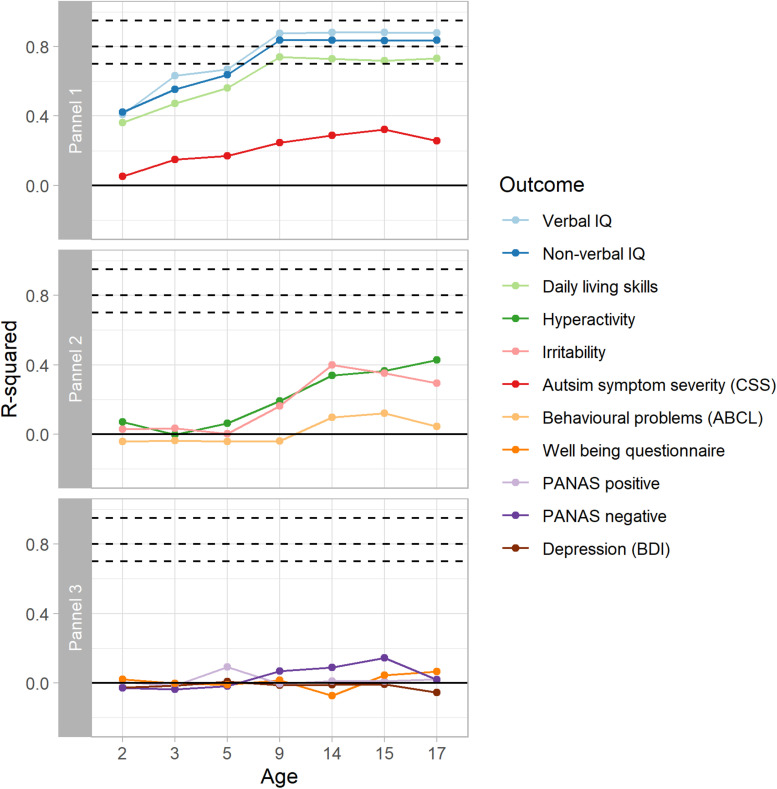
Optimism corrected predictive performance for continuous outcomes modeled using Lasso regression. Dashed lines show limits of predictive performance for test-retest ICCs of 0.95, 0.8, and 0.7. For verbal IQ, non-verbal IQ, and daily living, the results reach close to the test-retest limits for the outcome measures.

Figure 2 and Table 2 show predictive performance measured using R-squared for continuous outcomes modeled using lasso regression. Results from linear regression were similar and can be found in Supplementary Table 7. Test-retest of a measure places a ceiling on our ability to predict it, and meaningful prediction of a wholly unreliable measure is impossible. Unfortunately, we know little about the test-retest performance of many of these measures in samples of this kind, which for many characteristics can vary with age (Rinaldi and Karmiloff-Smith, 2017) and, especially for episodic depression, the performance will vary strongly with the interval between test times. For reference, Figure 2 therefore displays the upper limits on prediction for different test-retest intra-class correlations (ICC’s).

**TABLE 2:** Optimism corrected predictive performance for continuous outcomes, modeled using lasso regression.

Outcome	2	3	5	9	14	14*	15	17	17*
Verbal IQ	0.41	0.63	0.67	0.88	0.88	0.88	0.88	0.88	0.88
Non-verbal IQ	0.42	0.55	0.64	0.84	0.84	0.85	0.83	0.84	0.83
Daily living skills	0.36	0.47	0.56	0.74	0.73	0.74	0.72	0.73	0.75
Hyperactivity	0.07	0	0.06	0.19	0.34	0.37	0.37	0.43	0.43
Irritability	0.03	0.03	0	0.16	0.4	0.35	0.35	0.29	0.28
Autism symptom severity	0.05	0.15	0.17	0.25	0.29	0.23	0.32	0.26	0.25
Behavioral problems (ABCL)	−0.04	−0.04	−0.04	−0.04	0.1	0.07	0.12	0.04	0.05
Well-being questionnaire	0.02	0	−0.01	0.01	−0.07	0.1	0.04	0.06	0.21
PANAS positive	0.01	−0.02	0.09	0	0.01	0.08	0.01	0.02	−0.04
PANAS negative	−0.03	−0.04	−0.02	0.07	0.09	0.05	0.14	0.02	0.06
Depression (BDI)	−0.03	−0.02	0.01	−0.01	−0.01	−0.02	−0.01	−0.06	−0.04

*At age 14 and 17 additional analysis were conducted including the pro-social and emotion subscales from the strength’s and difficulties questionnaire.

The first panel in Figure 2 shows results for adult outcomes which were also assessed in early childhood, between ages 2 and 9. For verbal IQ, non-verbal IQ, and daily living skills, predictive performance increases from age 2 to age 9, when the optimism corrected R-squared was 0.88, 0.84, and 0.74, respectively, indicating that precise predictions can be made. The predictive performance at age 9 approaches the test-retest limit of 0.95 for verbal and non-verbal IQ and daily living skills (Balboni et al., 2016; Rinaldi and Karmiloff-Smith, 2017). In contrast to this, predicting autism symptom severity is much more challenging, with only small improvement with age. Our ability to predict adult autism symptom severity improves past age 9 with the addition of adolescent behavioral measures to the model but remains modest.

We found no ability to predict adult behavioral outcomes, such as irritability or hyperactivity from verbal and non-verbal IQ, daily living skills or autism symptom severity measured in childhood (Figure 2, panel 2). From age 9 into adolescence, there is an improvement in predictions of irritability (maximum R-squared 0.40, age 14) and hyperactivity (maximum R-squared 0.43, age 17) due to inclusion of measures in these same domains among the set of predictors that became available as part of the adolescent measurement batteries. Prediction for these outcomes remained modest and fell below what might be the expected test-retest limit. Our success in predicting behavioral problems measured using the ABCL total score (maximum R-squared 0.16) fell below even the modest success that we had with irritability and hyperactivity despite the total score from the related CBCL being included as a predictor in the model. The final panel of Figure 2 shows that we had minimal success in predicting positive or negative affect (maximum R-squared 0.08 and 0.14, respectively), depression (maximum R-squared 0.01) or well-being (maximum R-squared 0.21) from the data at any age. Inclusion of teacher-reports of behavioral and emotional problems measured using subscales of the SDQ at ages 14 or 17 did not lead to improvement in predictions for any outcome (Table 2).

**FIGURE 3:**
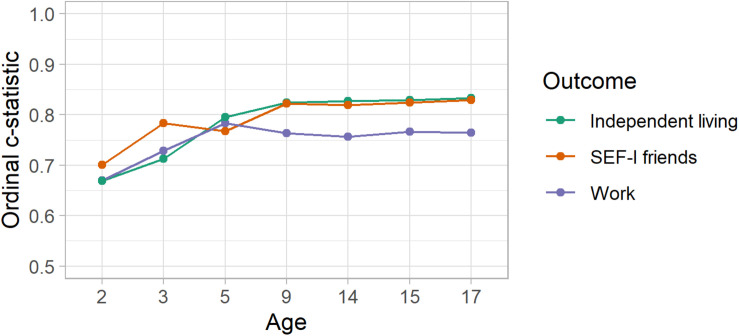
Optimism corrected predictive performance for ordinal outcomes.

Figure 3 and Table 3 shows the results for ordinal measures of work, independent living and the SEF-friends scales with improved prediction from age 2 to age 9 as updated measures of verbal and non-verbal IQ, daily living skills and autism symptom severity were added to the model (c-statistics at age 9 of for work, 0.76, independent living and 0.82, friends 0.82 indicating good model performance). There was no improvement in discriminative performance with inclusion of behavioral measures made after age 9.

**TABLE 3:** Optimism corrected performance for ordinal outcomes modeled using the proportional odds model.

Outcome	2	3	5	9	14	14*	15	17	17*
Independent living	0.67	0.71	0.79	0.82	0.83	0.81	0.83	0.83	0.82
SEF-I friends	0.7	0.78	0.77	0.82	0.82	0.83	0.82	0.83	0.82
Work	0.67	0.73	0.78	0.76	0.76	0.76	0.77	0.76	0.75

*At age 14 and 17 additional analysis were conducted including the 5 subscales from the strength’s and difficulties questionnaire.

### Parental Priorities

**FIGURE 4:**
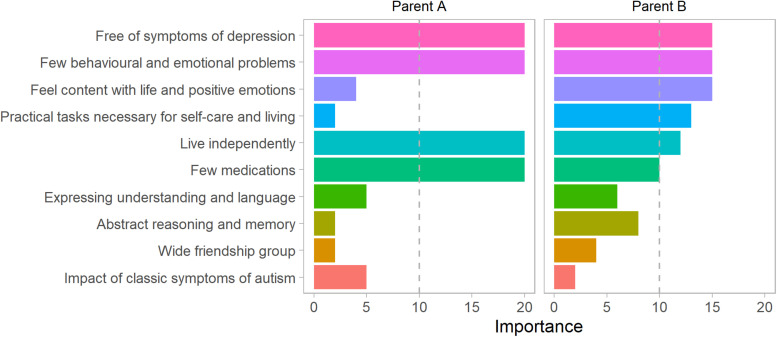
Individual parent priority profiles for two parents. This figure shows outcome priorities elicited from two parents of autistic children, labeled parent **(A)** and **(B)**. The dashed vertical line shows the priority that would be given if all outcomes were considered equal.

Figure 4 shows the priority profiles for two parents whose priorities we have used to demonstrate our methods for weighted predictions. Priorities greater than 10 indicate a question is given greater importance than if all items were considered equal. Areas of greater importance were depression, behavioral and emotional problems, contentedness and positive emotions. Least priority was given to questions relating to the classic symptoms of autism and having a wide friendship group.

### Personalized Composite Outcomes

**FIGURE 5:**
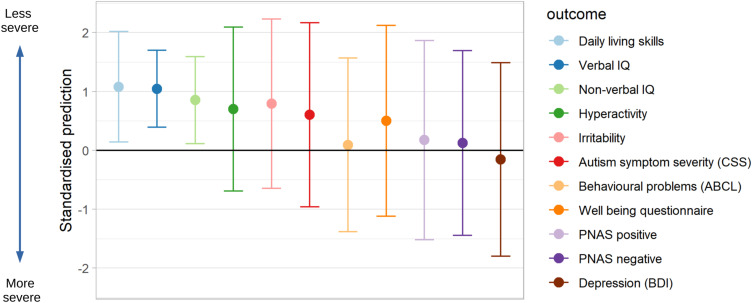
Predictions of continuous outcomes for a child, age 9, at 25th centile for impact of autism. Point predictions and 95% prediction intervals for a child with scores at the 25th centile for impact of ASD on predictors (Age 9 Verbal IQ = 101, Non-verbal IQ = 95, daily living skills = 45, CSS = 4, Age 14 irritability = 1, hyperactivity = 4, Age 15 CBCL = 50). Higher scores indicate a less severe impact of ASD.

Personalized composite outcomes were created by summing individual outcomes each with a weight. The weights were derived from the parent priorities questionnaire results. Figure 5 showed predictions, and 95% prediction intervals for a hypothetical child, aged 15, with scores across all predictors at the 25th centile for impact of autism of the imputed data used to fit the model (Age 9, Verbal IQ = 101, Non-verbal IQ = 95, daily living skills = 45, autism symptom severity (CSS) = 4, Age 14 irritability = 1, hyperactivity = 4, Age 15, CBCL = 50). The predictions of individual outcomes (Figure 5) showed that across the IQ measures and daily living skills it was most likely that the child would be less impacted by symptoms of autism in adulthood. Predictions for irritability, hyperactivity, and autism symptom severity showed that while it was likely that the impact would be less severe, there was still the possibility of a more profound effect of autism symptoms. For other outcomes, predictions close to zero and wide prediction intervals showed that predictions added little beyond describing the distribution in the population. These are in line with what we expected from our assessments of predictive performance.

**FIGURE 6:**
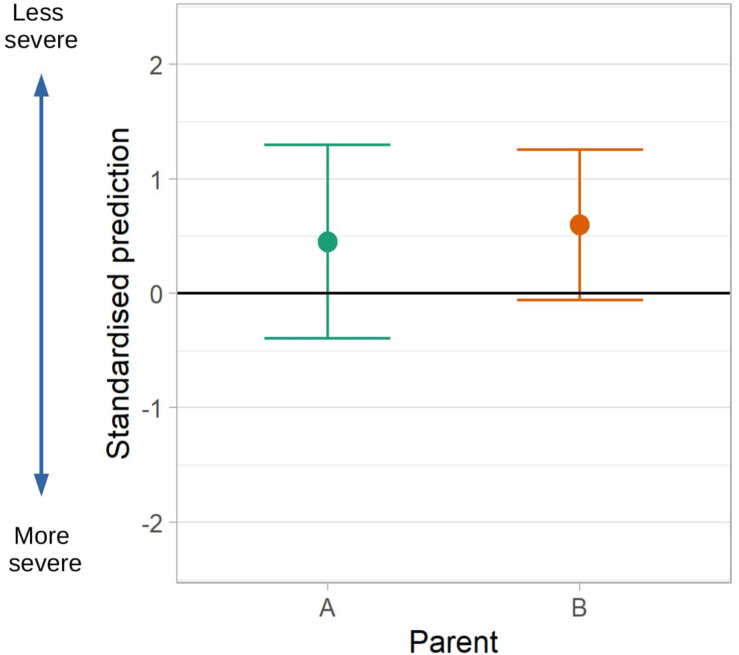
Predictions of a composite outcome formed with different parent priority profiles. Point predictions and 95% intervals for a child with scores at the 25th centile for impact of ASD on predictors (Age 9 Verbal IQ = 101, Non-verbal IQ = 95, daily living skills = 45, CSS = 4, Age 14 irritability = 1, hyperactivity = 4, Age 15 CBCL = 50). Higher scores indicate a less severe impact of ASD. Predictions of the composite outcome are similar for both parents, however based on Parent **(A)** priorities there is more certainty in the predictions than for Parent **(B)**.

Figure 6 showed predictions for a parent-specific composite outcome formed by weighting the same set of predictions of individual outcomes by the priority sets of different parents. Differing parent priorities led to different predictions of their composite with varying levels of confidence in prediction. For Parent A who placed relatively more weight on depression and well-being, areas that we were unable to predict well, the prediction interval (-1.30 to 0.4) was wide, and included some possibility of a greater than average impact of autism symptoms. For Parent B the 95% prediction interval (-1.26 to 0.06) largely excluded the possibility of a more severe than average impact of symptoms because Parent B placed greatest priority on items related to behavioral and emotional problems and practical tasks, for which by age 15 we achieved better prediction performance.

## Discussion

Discussions that patients, parents and clinicians have about priorities and planning in autism vary greatly. The structured evidence that we have for what is and what is not predictable is poor, leaving clinicians to make judgements based on their personal experience. Personal communication from some parents suggest that there may be a small minority of clinicians who feel very confident in what should be considered important, their ability to predict these, and who delivers their predictions with apparent certainty. Perhaps the principal message of this paper is that there is not necessarily the evidence to support such a practice as there can be differences in what parents consider important, and for some aspects of the adult outcome, prediction can be extremely challenging.

Based on assessments of verbal and non-verbal IQ, daily living skills and autism symptom severity it is possible to make good predictions at age 9 of a child’s adult IQ and adaptive functioning. Predictions of friends, work and living situation are also possible. The importance of verbal and non-verbal IQ and symptom severity in making long term prognosis for autistic children is consistent with results from previous systematic reviews (Magiati et al., 2014; Zimmerman et al., 2018). However, these measures are insufficient to predict adult assessments of irritability and hyperactivity, and it is only with the inclusion of adolescent measures of these outcomes that predictions of these outcomes are possible with modest certainty. No measurements of irritability or hyperactivity were made prior to age 9, however, the relatively poor predictions possible with assessment of these measures at age 9 indicate earlier assessments would be unlikely to contribute to improved predictions. Predictions of the severity of autism symptoms measured using the CSS improved as childhood measures of IQ, daily living skills and autism symptom severity were updated, and with the addition of behavioral measures made in adolescence. Predicting other measures which make up the adult outcome proved extremely challenging. Based on the available data it is not possible to predict behavioral problems measured using the ABCL, adult well-being, depression, or positive or negative emotions with any certainty. While it may be considered that accurate prediction is the critical goal for planning, providing an evidence-based indication of the uncertainty of predictions is equally important. This avoids unsubstantiated over-generalized and deterministic views of the future, maintaining scope for appropriate hopes and ambitions while avoiding both the dispiriting effects of failing to achieve near-certain unrealistically positive goals and failing to grasp within-range opportunities through unjustified pessimism.

Predictions of the composite of all outcomes formed using parental priorities are more precise and have prediction intervals that encompass a range of outcomes closer to the average than predictions of the individual components. This is due to the weak correlation among the prediction errors across the outcome profile. This leads to predictions closer to the average because a more- extreme- than- expected- outcome in one aspect of the outcome profile does not mean that a more- extreme- outcome should be expected in another aspect of the profile. Therefore, when combining predictions for many outcome measures, a less extreme outcome will be predicted.

The priorities elicited from parents show that it is possible to incorporate these views into predictions of future outcomes. Although not generalisable to the wider population, the parent priorities elicited in this study show that there is diversity in what parents of autistic children consider important in relation to adult outcomes. This is consistent with observations that what makes up a good outcome may be personal (Lounds Taylor, 2017). The aspects of the outcome related to symptoms of depression, contentedness with life and positive emotion, where we had the greatest challenges in making predictions, were amongst the areas given highest priority on average by parents. There is no need to assume that the that priorities need be fixed for a particular child. It would be very reasonable to expect priorities to reflect current concerns more sharply than those associated with a hard to imagine domain of a distant future life. While recalculation and discussion with updated weights may be appropriate, consideration might be given to a series of such discussions over the years, but with each being concerned with outcomes more proximal in time and developmentally closer to the contemporaneous lived experience.

The models developed in this study are highly informative with regard to how different facets of the adult outcome can be predicted and how predictions change over time, but further external validation, in new data, is required before the results should be used to make personalized predictions in clinical practice (Steyerberg and Harrell, 2016). The modest sample size available in this study also means that there was the potential for overfitting. This could have led to optimistic estimates of model performance for irritability, hyperactivity and autism symptom severity (measured using the CSS). The risk of overfitting from more complex models also lead to us adopting a conservative analysis strategy, using a relatively simple modeling approach. A larger sample size may support more complex modeling which could improve prediction accuracy. The findings are based on a single study, carried out in a particular context; predictive performance in areas we found it to be poor may improve if additional measures were included in childhood or adolescence. A change in the availability of effective therapies could also lead to different results. The modeling approach we took means our results should be interpreted in the context of a child undergoing two assessments—one between the age of 2 and 9, and a second in adolescence. A different approach, incorporating repeated measures of the same predictor in models would be required to model the effect of a more intense program of assessment.

Future work should consider replicating these findings in other datasets, investigating whether different sets of measurements can improve predictions in areas we found challenging, and considering analysis approaches which incorporate repeated measures of predictors. Further development of prediction models would also benefit from a participatory approach where autistic people and parents of autistic children are involved in all stages of the research. Predictions might also change as the life-opportunities of autistic adults change, given substantial geographical and temporal variability. Additional work is also clearly required to better understand what parents of autistic children, and the children themselves, want and want to know. In addition, differences in priorities between autistic individuals, their families, and the professionals they work with, and for children of differing capabilities, developmental stage and in different settings of cultural expectations and opportunity could be examined.

## Conclusion

Assessments in childhood can lead to good predictions of cognitive ability, daily living skills, and social functioning. Predictions improve with age up to age 9. Prediction of the severity of autism symptoms in adulthood improved throughout childhood and adolescence, but predictions remained weaker than for cognitive ability or adaptive functioning. For behavioral aspects of the adult outcome, prediction is only possible with assessments made in adolescence and even then remain uncertain. For aspects of the adult outcome relating to mental health and well-being, prediction was extremely difficult at any age. One feasible way to summarize multi-faceted adult outcomes is to combine different adult outcomes measures into a single composite based on the individual or consensus priorities of parents, clinician and autistic individuals. We are continuing to work on the development of methodology and tools that can facilitate the process of discussing the future and its implications for current priorities and planning.

## Data Availability Statement

The datasets presented in this article are not readily available because they contain personal sensitive data concerning the health of participants. Requests to access the datasets should be directed to GF, gordon.forbes@kcl.ac.uk.

## Ethics Statement

The Early Diagnosis Cohort was reviewed and approved by the Weill Cornell Medicine IRB and IRBs at the University of Michigan. Written informed consent to participate in this study was provided by the participants’ legal guardian/next of kin.

## Author Contributions

GF conducted the statistical analysis, wrote the first draft of the manuscript. AP conceived the idea for the analysis provided input into methods used. CL has led the study since its inception and RE lead the data collection in adulthood. All authors contributed to further drafts of the manuscript.

## Conflict of Interest

CL acknowledges the receipt of royalties from the sale of the Autism Diagnostic Observation Schedule (ADOS) and the Autism Diagnostic Interview-Revised (ADI-R). Royalties generated from this study were donated to a not-for-profit agency, Have Dreams. The remaining authors have declared that they have no competing or potential conflicts of interest.
