# Supplementary material for: Predicting Uncertain Multi-Dimensional Adulthood Outcomes From Childhood and Adolescent Data in People Referred to Autism Services
Source: Front Psychol. 2021 Feb 9;12:594462. doi: 10.3389/fpsyg.2021.594462 (PMC7900001; doi:10.3389/fpsyg.2021.594462)

***Predicting Uncertain Multi-Dimensional Adulthood Outcomes From Childhood and Adolescent Data in People Referred to Autism Services***

Supplementary Material

**Contents**

[Supplementary table 1: Schedule of assessment for measures included in this study. 3](#_Toc62140943)

[Supplementary table 2: Measures. 4](#_Toc62140944)

[Supplementary table 3: Parent priorities questionnaire 5](#_Toc62140945)

[Supplementary table 4: Mapping of priorities questionnaire to outcomes 5](#_Toc62140946)

[Supplementary table 5: Predictors included at each timepoint 6](#_Toc62140947)

[Supplementary table 6: Descriptive statistics for measures at all timepoints 7](#_Toc62140948)

[Supplementary table 7: Optimism corrected predictive performance for continuous outcomes, modelled using linear regression 9](#_Toc62140949)

[Supplementary Figure 1. Optimism corrected predictive performance for continuous outcomes (Linear regression) 10](#_Toc62140950)

### Supplementary table 1: Schedule of assessment for measures included in this study.

|  | Age | | | | | | | |
| --- | --- | --- | --- | --- | --- | --- | --- | --- |
| **Measure** | **2** | **3** | **5** | **9** | **14** | **15** | **17** | **Adulthood** |
| CSS | x | x | x | x |  |  |  | x |
| Verbal IQ | x | x | x | x |  |  |  | x |
| Non Verbal IQ | x | x | x | x |  |  |  | x |
| Diagnosis of ASD | x | x | x | x |  |  |  |  |
| Daily living | x | x | x | x |  |  |  | x |
| Irritability |  |  |  | x | x |  | x | x |
| Hyperactivity |  |  |  | x | x |  | x | x |
| CBCL |  |  |  | x |  | x |  | x |
| SDQ |  |  |  |  | x |  | x |  |
| BDI |  |  |  |  |  |  |  | x |
| PANAS-N |  |  |  |  |  |  |  | x |
| PANAS-P |  |  |  |  |  |  |  | x |
| WBQ |  |  |  |  |  |  |  | x |
| Independent Living |  |  |  |  |  |  |  | x |
| Work |  |  |  |  |  |  |  | x |
| SEF-friends |  |  |  |  |  |  |  | x |

### Supplementary table 2: Measures.

| **Measure Name** | **Acronym** | **Citation** | **Measured Construct(s)** | **Range and Interpretation** |
| --- | --- | --- | --- | --- |
| Aberrant Behavior Checklist | ABC | Aman, Singh, Stewart, & Field, 1985 | Irritability (IRRIT) & Hyperactivity (HYP) | IRRIT: 0-45  HYP: 0-48;  Higher scores indicate more severe problems |
| Adult/Child Behavior Checklist | ABCL Total  CBCL | Achenbach, & Rescorla, 2006 | Total Behavior Problems | ABCL Total: 20-100  CBCL Total: 20-100  Higher scores indicate more severe problems |
| Autism Diagnostic Observation Schedule 2 | ADOS-2 | Lord et al.., 2012 | Comparative Severity Score (CSS) | CSS: 1-10  Higher scores indicate higher autism severity |
| Beck Depression Inventory-II | BDI | Beck, Steerm & Brown, 1996 | Depressive symptoms | BDI: 0-30  Higher scores indicate higher symptoms |
| Positive and Negative Affect Scale | PANAS-P;  PANAS-N | Watson, Clark, & Tellegen, 1988 | Levels of positive or  negative affect | PANAS-P: 0-40  PANAS-N: 0-40  Higher scores indicate higher affect states |
| Social Emotional Functioning Interview | SEF-I | Rutter, et al., 1988 | Work, Living, Friendships | Work: 1-7  Living: 1-3  Friends: 0-3 |
| Strength & Difficulties | SDQ | Goodman (1996) | Conduct, Emotion, Hyperactivity, peer relationships, pro-social. | All scales 5-15, |
| VABS | DLS | Sparrow et al., 2005 | Daily Living Skills | DLS: 17-112  Higher scores indicate greater adaptive skills |
| Wellbeing Scale | WBQ | Ryff 1989 | Psychological Wellbeing | 134-248  Higher scores indicate higher wellbeing |

### Supplementary table 3: Parent priorities questionnaire

| **Instructions**  You have 100 points to allocate to express how important you consider the following domains to be in achieving the best adult outcome for your child.  You must allocate allocate all 100 points but no more. The total number of points allocated is shown at the bottom of the questions. For example, if you consider each of the domains to be of equal importance give each domain 10 points. |
| --- |
| 1. I would like my child to be less impacted by the classic symptoms of autism 2. I would like my child to be skilled in expressing and understanding language 3. I would like my child to be skilled in abstract reasoning and memory tasks 4. I would like my child to be able to live independently with as little family or official support as possible 5. I would like my child to have a wide friendship group 6. I would like my child to feel content with life and experience positive emotions. 7. I would like my child to be free of symptoms of depression such as low mood, negative affect, worry and tiredness. 8. I would like my child to be able to carry out all the practical tasks necessary for self-care and living. 9. I would like my child to have as few behavioral and emotional problems as possible.   (10) I would like my child to be able to manage with as few medications as possible. |

### Supplementary table 4: Mapping of priorities questionnaire to outcomes

| Question | Outcome(s) |
| --- | --- |
| **Practical tasks necessary for self-care and living** | Daily living |
| **Expressing understanding and language** | Verbal IQ |
| **Abstract reasoning and memory** | Non-verbal IQ |
| **Few behavioural and emotional problems** | Hyperactivity, Irritability, CBCL |
| **Free of symptoms of depression** | Depression (BDI), PNAS negative |
| **Feel content with life and positive emotions** | PNAS positive, Wellbeing questionnaire |
| **Live independently** | Work, Independent living |
| **Wide friendship group** | SEF-friends |
| **Impact of classic symptoms of autism** | CSS |

### Supplementary table 5: Predictors included at each timepoint

| **Age/Analysis** | **New measures included** |
| --- | --- |
| Demographic characteristics included in all analysis | Race, mother’s education, and gender. |
| 2 | Age 2 assessments of verbal IQ, non-verbal IQ, CSS, daily living, estimated diagnosis |
| 3 | Age 3 Verbal IQ, non-verbal IQ, Comparative severity score, daily living skills, estimated diagnosis |
| 5 | Age 5 Verbal IQ, non-verbal IQ, Comparative severity score, daily living skills, estimated diagnosis |
| 9 | Age 9 Verbal IQ, non-verbal IQ, Comparative severity score, daily living skills, estimated diagnosis, Irritability, Hyperactivity, and CBCL |
| 14 | Age 9 verbal IQ, non-verbal IQ, Comparative severity score, daily living skills, estimated diagnosis, CBCL  Age 14 Irritability, hyperactivity |
| 14* | Age 9 verbal IQ, non-verbal IQ, Comparative severity score, daily living skills, estimated diagnosis, CBCL  Age 14 Irritability, hyperactivity, SDQ-prosocial, SDQ-emotion |
| 15 | Age 9 verbal IQ, non-verbal IQ, Comparative severity score, daily living skills, estimated diagnosis  Age 14 Irritability, hyperactivity, SDQ-prosocial, SDQ-emotion  Age 15 CBCL |
| 17 | Age 9 verbal IQ, non-verbal IQ, Comparative severity score, daily living skills, estimated diagnosis  Age 15 CBCL  Age 17 Irritability, hyperactivity, SDQ-prosocial, SDQ-emotion |
| 17* | Age 9 verbal IQ, non-verbal IQ, Comparative severity score, daily living skills, estimated diagnosis  Age 15 CBCL  Age 17 Irritability, hyperactivity, SDQ-prosocial, SDQ-emotion |

### Supplementary table 6: Descriptive statistics for measures at all timepoints

| Measure | Summary | 2 | 3 | 5 | 9 | 14 | 15 | 17 | Adult |
| --- | --- | --- | --- | --- | --- | --- | --- | --- | --- |
| **Autism Symptom severity (CSS)** | N | 82 | 77 | 63 | 96 | - | - | - | 118 |
|  | Median (IQR) | 8 (6, 9) | 7 (5, 8) | 6 (3, 8) | 7 (4, 9) | - | - | - | 6 (3, 7) |
|  | Range | 1-10 | 1-10 | 1-10 | 1-10 | - | - | - | 1-10 |
| **Verbal IQ** | N | 104 | 92 | 72 | 112 | - | - | - | 123 |
|  | Median (IQR) | 32 (22, 54) | 46 (26, 74) | 58 (34, 80) | 55 (28, 92) | - | - | - | 46 (20, 104) |
|  | Range | 10-108 | 10-110 | 12-105 | 3-140 | - | - | - | 2-139 |
| **Non-verbal IQ** | N | 104 | 91 | 71 | 112 | - | - | - | 123 |
|  | Median (IQR) | 72 (52, 84) | 68 (48, 82) | 70 (50, 90) | 67 (39, 94) | - | - | - | 72 (26, 105) |
|  | Range | 13-132 | 17-120 | 14-131 | 2-131 | - | - | - | 3-133 |
| **Daily living skills** | N | 104 | 91 | 63 | 97 | - | - | - | 123 |
|  | Median (IQR) | 68 (62, 74) | 64 (57, 68) | 64 (47, 75) | 40 (19, 72) | - | - | - | 61 (36, 78) |
|  | Range | 52-99 | 47-94 | 35-110 | 17-109 | - | - | - | 17-112 |
| **Hyperactivity** | N | - | - | - | 71 | 75 | - | 77 | 104 |
|  | Median (IQR) | - | - | - | 10 (5, 16) | 8 (4, 15) | - | 7 (2, 15) | 5 (2, 12) |
|  | Range | - | - | - | 0-40 | 0-31 | - | 0-37 | 0-31 |
| **Irritability** | N | - | - | - | 71 | 75 | - | 77 | 104 |
|  | Median (IQR) | - | - | - | 6 (1, 14) | 5 (1, 13) | - | 5 (1, 12) | 5 (1, 10) |
|  | Range | - | - | - | 0-32 | 0-29 | - | 0-36 | 0-38 |
| **SDQ emotion** | N | - | - | - | - | 65 | - | 49 | - |
|  | Median (IQR) | - | - | - | - | 7 (6, 9) | - | 7 (5, 8) | - |
|  | Range | - | - | - | - | 5-13 | - | 5-12 | - |
| **SDQ pro-social** | N | - | - | - | - | 56 | - | 43 | - |
|  | Median (IQR) | - | - | - | - | 9 (6, 12) | - | 10 (7, 12) | - |
|  | Range | - | - | - | - | 5-15 | - | 5-15 | - |
| **CBCL/ABCL** | N | - | - | - | 68 | - | 74 | - | 94 |
|  | Median (IQR) | - | - | - | 58 (50, 67) | - | 56 (49, 61) | - | 53 (48, 57) |
|  | Range | - | - | - | 33-88 | - | 24-73 | - | 25-77 |
| **Wellbeing questionnaire** | N | - | - | - | - | - | - | - | 91 |
|  | Median (IQR) | - | - | - | - | - | - | - | 189 (169, 208) |
|  | Range | - | - | - | - | - | - | - | 134-248 |
| **PANAS-P** | N | - | - | - | - | - | - | - | 92 |
|  | Median (IQR) | - | - | - | - | - | - | - | 28 (23, 34) |
|  | Range | - | - | - | - | - | - | - | 12-46 |
| **PANAS-N** | N | - | - | - | - | - | - | - | 93 |
|  | Median (IQR) | - | - | - | - | - | - | - | 15 (12, 21) |
|  | Range | - | - | - | - | - | - | - | 10-36 |
| **Depression (BDI)** | N | - | - | - | - | - | - | - | 92 |
|  | Median (IQR) | - | - | - | - | - | - | - | 2 (0, 7) |
|  | Range | - | - | - | - | - | - | - | 0-30 |
| **Living** | N | - | - | - | - | - | - | - | 123 |
|  | Median (IQR) | - | - | - | - | - | - | - | 2 (2, 2) |
|  | Range | - | - | - | - | - | - | - | 1-3 |
| **SFI-friends** | N | - | - | - | - | - | - | - | 106 |
|  | Median (IQR) | - | - | - | - | - | - | - | 2 (0, 3) |
|  | Range | - | - | - | - | - | - | - | 0-3 |
| **Work** | N | - | - | - | - | - | - | - | 113 |
|  | Median (IQR) | - | - | - | - | - | - | - | 4 (2, 6) |
|  | Range | - | - | - | - | - | - | - | 1-7 |

### Supplementary table 7: Optimism corrected predictive performance for continuous outcomes, modelled using linear regression

| Outcome | 2 | 3 | 5 | 9 | 14 | 14* | 15 | 17 | 17* |
| --- | --- | --- | --- | --- | --- | --- | --- | --- | --- |
| **Verbal IQ** | 0.4 | 0.62 | 0.65 | 0.86 | 0.87 | 0.88 | 0.87 | 0.87 | 0.87 |
| **Non-verbal IQ** | 0.41 | 0.56 | 0.64 | 0.83 | 0.83 | 0.83 | 0.83 | 0.83 | 0.83 |
| **Daily living skills** | 0.33 | 0.46 | 0.54 | 0.73 | 0.74 | 0.74 | 0.74 | 0.74 | 0.74 |
| **Hyperactivity** | -0.01 | 0.03 | 0.04 | 0.18 | 0.38 | 0.39 | 0.37 | 0.42 | 0.39 |
| **Irritability** | -0.04 | -0.04 | -0.06 | 0.11 | 0.37 | 0.37 | 0.33 | 0.3 | 0.25 |
| **Autism symptom severity** | 0.05 | 0.13 | 0.16 | 0.21 | 0.24 | 0.21 | 0.3 | 0.23 | 0.17 |
| **Behavioral problems (ABCL)** | -0.13 | -0.24 | -0.12 | 0.07 | 0.12 | 0.16 | 0.08 | 0.07 | -0.03 |
| **Wellbeing questionnaire** | -0.13 | -0.12 | -0.11 | -0.11 | -0.17 | -0.11 | -0.1 | -0.01 | 0 |
| **PANAS positive** | -0.11 | -0.14 | -0.03 | -0.13 | -0.15 | -0.15 | -0.16 | -0.13 | -0.02 |
| **PANAS negative** | -0.18 | -0.18 | -0.21 | -0.06 | 0.16 | 0.13 | 0.18 | 0 | -0.16 |
| **Depression (BDI)** | -0.12 | -0.12 | -0.14 | -0.16 | -0.16 | -0.24 | -0.05 | -0.04 | -0.14 |

*At age 14 and 17 additional analysis were conducted including the 5 subscales from the strength’s and difficulties questionnaire and the impact score.

### Supplementary Figure 1. Optimism corrected predictive performance for continuous outcomes (Linear regression)

Dashed lines show limits of predictive performance for test retest ICCs of 0.95, 0.8, and 0.7


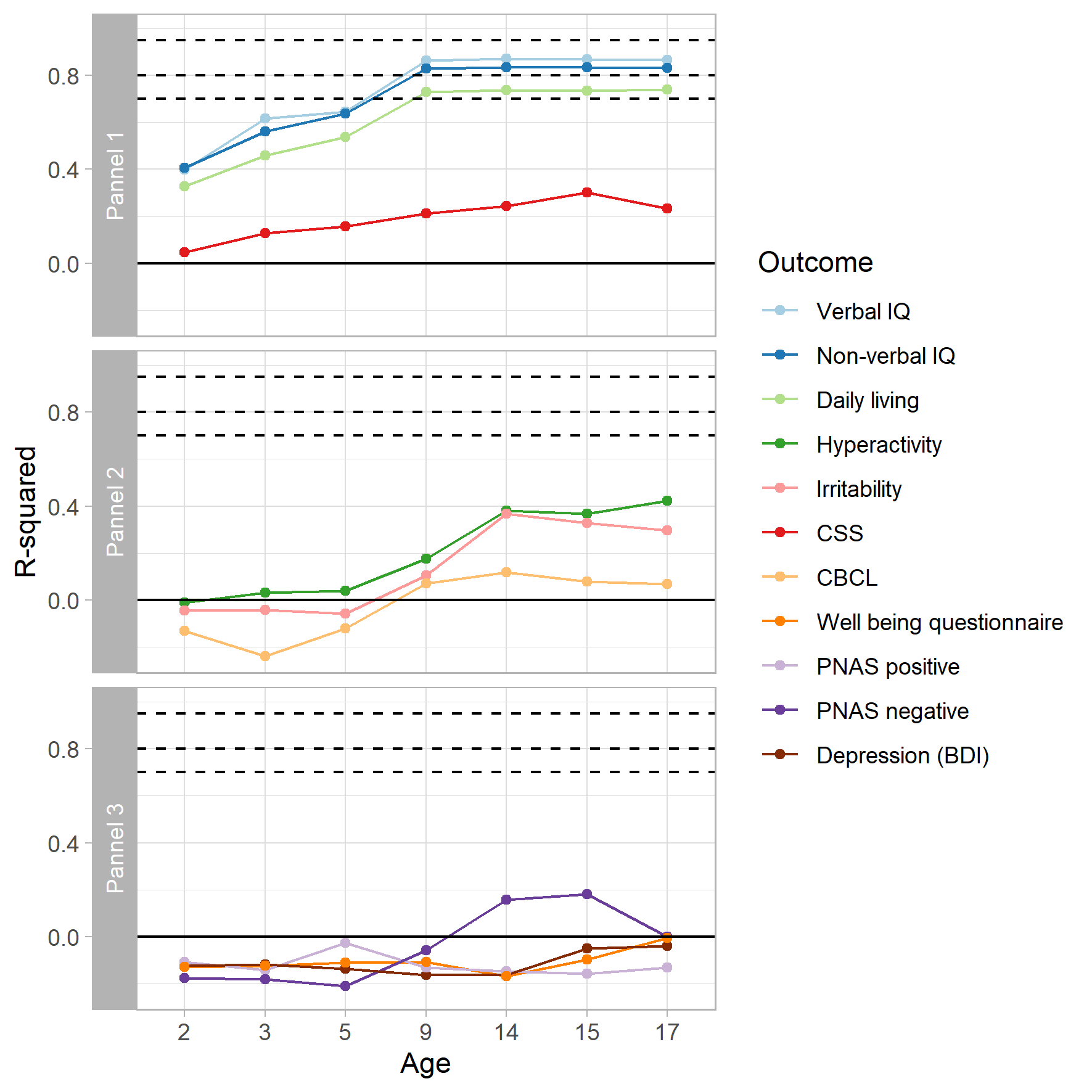

Supplement: Supplementary file 1 [file Table_1.docx]
